# Bilateral asteroid hyalosis revealing a blood imbalance

**DOI:** 10.11604/pamj.2013.16.87.3329

**Published:** 2013-11-09

**Authors:** Zouheir Hafidi, Rajae Daoudi

**Affiliations:** 1 Université Mohammed V Souissi, Service d'Ophtalmologie A de l'hôpital des spécialités, Centre Hospitalier Universitaire, Rabat, Maroc

**Keywords:** Asteroid hyalosis, vitreous degeneration, retinopathy, fluorescein angiography

## Image in medicine

Asteroid hyalosis is an age related vitreous degeneration of unknown etiology, usually described to be unilateral. It's characterized by aggregation of calcium soaps in vitreous body. This benign condition has been reported to be frequently associated to many systemic disorders including diabetes mellitus, systemic arterial hypertension, atherosclerotic vascular disease, hypercholesterolemia and increased serum calcium levels. We report an unusual case of bilateral asteroid hyalosis revealing a diabetes in a previously healthy man. A 65-year old man presented with 2 years history of increasing bilateral eye floaters. The best corrected visual acuity was 6/10 in the right eye and 3/10 in the left eye. At examination there were dense vitreous clouds of bright white particles in both eyes, with fundus-obscuring; particularly in the left eye (Panel A), an abnormality of the retinal vasculature was suspected in the right eye (arrow). Fluorescein angiography showed bilateral retinal neovascularization (Panel B, arrowheads). A workup was performed revealing high levels of fasting glucose and glycohemoglobin with high levels of serum calcium, arterial pressure was within normal limits. This presentation was consistent with proliferative diabetic retinopathy with bilateral asteroid hyalosis. The patient was then referred to department of diabetology for further investigations and management of his diabetes. Meanwhile, Laser retinal photocoagulation was started in the right eye. However the marked vitreous degeneration in the left eye made photocoagulation with a conventional contact lens difficult; thus, prompt posterior vitrectomy with endolaser photocoagulation was indicated after intravitreal injection of anti-endothelial growth factor (anti VEGF) agents.

**Figure 1:**
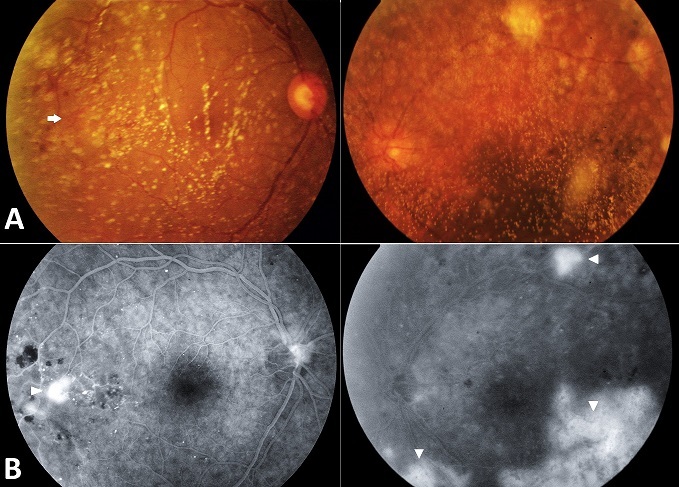
A) Photograph of fundus showing dense vitreous clouds of bright white particles in both eyes with fundus-obscuring, particularly in the left eye, with retinal neovascularization in the right eye (arrow); B) angiogram showing retinal neovascularization in both eyes (arrowheads)

